# Computationally Efficient Concept of Representative Directions for Anisotropic Fibrous Materials

**DOI:** 10.3390/polym14163314

**Published:** 2022-08-15

**Authors:** Alexey Shutov, Alexander Rodionov, Dmitri Ponomarev, Yana Nekrasova

**Affiliations:** 1 Lavrentyev Institute of Hydrodynamics, Pr. Lavrentyeva 15, 630090 Novosibirsk, Russia; 2 Saint Petersburg State Marine Technical University, Ul. Lotsmanskaya 3, 190121 Saint Petersburg, Russia

**Keywords:** fibrous composites, polymers, large strain, concept of representative directions, computational efficiency, electrospinning

## Abstract

The concept of representative directions allows for automatic generation of multi-axial constitutive equations, starting from simplified uni-axial material models. In this paper, a modification of the concept is considered suitable for the analysis of fibrous polymeric materials, which are anisotropic in the as-received state. The modification of the concept incorporates an orientation probability density function (OPDF), which explicitly accounts for the material anisotropy. Two versions of the concept are available. The first version utilizes the homogeneous distribution of the representative directions, with the entire anisotropy being contained in the weighting factors. The second encapsulates the anisotropy in the distribution of the representative directions. Due to its nature, the second version allows for a more efficient use of computational power. To promote this efficient version of the concept, we present new algorithms generating required sets of representative directions that match a given OPDF. These methods are based (i) on the minimization of a potential energy, (ii) on the equilibration method, and (iii) on the use of Voronoi cells. These three methods are tested and compared in terms of various OPDFs. The applicability of the computationally efficient modeling method to mechanical behavior of an anisotropic polymeric material is demonstrated. In particular, a calibration procedure is suggested for the practically important case when the OPDF is not known a-priori.

## 1. Introduction

One-dimensional material laws are often deduced by materials scientists, based on in-depth understanding of the underlying physical phenomena or by data-driven approaches [1,2]. These one-dimensional laws provide axial stresses as a function of the local history of axial strain. Unfortunately, such uni-axial models are not suitable for boundary value problems, neither can they be implemented into the finite element method for analysis of bulk structures. To enable finite element modeling, a number of procedures have been developed which generalize one-dimensional constitutive laws to new multi-axial material models. One such procedure is the concept of representative directions [3,4,5,6,7]. Within this concept, each material particle is associated with a collection of fibers, which we also call representative directions. The generalization of the material model is based on the postulate that the overall stress power is equal to the sum of the stress powers of individual fibers (directions). Note that in the special case of hyperelastic materials, this concept is equivalent to the integration approach [8], which is also called the “angular integration approach” [9,10,11,12,13,14]. The angular integration approach is based on the more specific postulate that the total potential energy is the sum of the energies stored in individual fibers. We emphasize that the concept of representative directions is more general since it does not require the existence of the potential energy of individual fibers.

Along with the concept of representative directions, there are similar approaches to material modeling that also lead to integration over the unit sphere. For example, the microplane approach proposed in [15], as well as the micro-sphere approach, see [16,17,18].

The current study uses the classical (affine) concept of representative directions as a starting point. Namely, we implement the concept based on the right Cauchy-Green tensor C; for brevity, we call this concept the C-approach. Only incompressible materials are modeled here. Nevertheless, the concept of representative directions can be easily generalized to predict the stress response in compressible materials as well, cf. [3,19].

The case of initially isotropic materials has been covered in numerous publications. In particular, it has been shown that the concept of representative directions can describe the load-induced anisotropy in such materials with a surprising accuracy [4]. The current study is devoted to a more general case of materials anisotropic in the as-received state, where the initial anisotropy is described by a proper orientation probability density function (OPDF). Dealing with fibrous materials, the OPDF provides information on how many fibers are aligned along a certain direction in the reference state. Despite its high generality, the straightforward use of OPDFs within the concept of representative directions is computationally expensive since it requires a tedious integration over the unit sphere. Therefore, in some applications, refined approaches are commonly implemented based on generalized structural tensors [20,21,22]. In the current study, we suggest another remedy to the problem of excessive computational costs.

The goal of this work is twofold. First, we propose a computationally efficient method based on the concept of representative directions. Its high efficiency is due to the use of heterogeneously distributed fibers (representative directions) matching a given OPDF. Second, we suggest three algorithms generating the required sets of fibers matching the given OPDF. As a byproduct, we also demonstrate the modeling chain for strongly anisotropic materials when the proper OPDF is not known a-priori.

This paper is organized as follows. In Section 2 we recall the main ingredients of the concept of representative directions. New algorithms are provided, generating sets of representative directions (fibers) matching a given OPDF. These algorithms are (i) the energy minimization (Landau–Ginzburg) method, (ii) the equilibration method, and (iii) the method of Voronoi cells. Various tests involving OPDFs of Mises–Fischer, Vallée-Poussin, and orthotropic OPDF based on a structural tensor were carried out for each of the proposed methods. In Section 3 we calibrate an orthotropic material model against actual testing data from [23]. After the calibration is complete, an anisotropic set of fibers is generated using the identified OPDF and a good correspondence between experiment and simulation is observed. Section 4 presents the discussion of the main results.

We conclude this introduction by a few words regarding notation. A coordinate-free tensor formalism described in [24] is applied in this study. Tensors of the first and second rank in $R^{3}$ are typed in bold. The symbol 1 denotes the identity tensor; $A^{D}:=A-\frac{1}{3}\mathrm{tr}\left(A\right)1$ is the deviatoric part of the tensor. The double contraction (scalar product) of two tensors is denoted as $A:B=\mathrm{tr}(A\cdot B^{T})$.

## 2. Modeling of Initially Anisotropic Materials

### 2.1. General Concept of Representative Directions

Let F be the deformation gradient tensor. The local strain history is given by the history of the right Cauchy–Green tensor $C\left(t\right)=F^{T}\left(t\right)F\left(t\right)$, t∈[0,T]. The classical concept of representative directions provides the second Piola–Kirchhoff stress tensor $\tilde{T}\left(t\right)$ as a function of the right Cauchy–Green tensor $C(t^{\prime })$, $t^{\prime }\in \left[0,t\right]$ [3,6]. Note that all models of this type are automatically objective [25].

Suppose that the material particle is idealized as a set of *N* fibers which we also call “representative directions”. In [17] and other studies, instead of the general term “fiber”, the authors prefer the micromechanically justified term “chain”. In addition, in the micro-sphere approach the models of individual chains are two-dimensional, whereas in our study the fibers are one-dimensional.

In the reference configuration, each fiber is uniquely determined by the unit vector $e_{\alpha }$, $∥e_{\alpha }∥=1$, α=1,2,…,N. The stretch of the fiber with the number α is calculated as

(1) $$\lambda_{\alpha }:=\sqrt{e_{\alpha }\cdot C\cdot e_{\alpha }}=\sqrt{C:(e_{\alpha }⊗e_{\alpha })}.$$

Suppose that for each fiber with the number α there is a one-dimensional material law. This law specifies the uniaxial stress $\sigma_{\alpha }\left(t\right)$ as a function of the history of the stretch $\lambda_{\alpha }\left(t^{\prime }\right)$, $t^{\prime }\in \left[0,t\right]$. It is important that the material law is the same for all fibers. A simple one-dimensional material law will be presented later on.

Within the concept of representative directions uniaxial stress $\sigma_{\alpha }$ is the stress which is power conjugate to the true strain $\varepsilon_{\alpha }=\ln\left(\lambda_{\alpha }\right)$. In other words, the stress power of the individual fiber is equal to the product $\sigma_{\alpha }\;{\dot{\varepsilon }}_{\alpha }$. Dealing with incompressible materials, the stress $\sigma_{\alpha }$ coincides with the true stress (also known as the Cauchy stress).

Consider the incompressibility condition:

(2) $$\det F\equiv 1\;\Rightarrow \;\det C\equiv 1\;\Rightarrow \;\dot{C}:C^{-1}=0.$$

From the basic assumption that the overall stress power of the material equals the weighted average of the stress powers of individual fibers, we can calculate the deviatoric part of the stress tensor. Indeed, the balance of stress powers is as follows:

(3) $$\tilde{T}:\frac{1}{2}\dot{C}=\sum_{\alpha =1}^{N}\omega_{\alpha }\sigma_{\alpha }\cdot \frac{d}{dt}\left(\ln\lambda_{\alpha }\right)\;\mathrm{for}\;\mathrm{all}\;\dot{C},\;\mathrm{such}\;\mathrm{that}\;\dot{C}:C^{-1}=0.$$

Here, $\tilde{T}$ is the second Piola–Kirchhoff stress tensor; $\omega_{\alpha }\geq 0$ are constant weighting coefficients corresponding to the fibers $e_{\alpha }$. Differentiating (1) with respect to time, we obtain the rate of the true strain:

(4) $${\dot{\varepsilon }}_{\alpha }=\frac{d}{dt}\left(\ln\lambda_{\alpha }\right)=\frac{1}{2\lambda_{\alpha }^{2}}\left(e_{\alpha }⊗e_{\alpha }\right):\dot{C}\;\mathrm{for}\;\mathrm{all}\;\alpha =1,2,\ldots ,N.$$

Substituting this kinematic expression into the power balance (3), we obtain

(5) $$\tilde{T}:\frac{1}{2}\dot{C}=\sum_{\alpha =1}^{N}\omega_{\alpha }\sigma_{\alpha }\cdot \frac{1}{2\lambda_{\alpha }^{2}}\left(e_{\alpha }⊗e_{\alpha }\right):\dot{C}\;\mathrm{for}\;\mathrm{all}\;\dot{C},\;\mathrm{such}\;\mathrm{that}\;\dot{C}:C^{-1}=0.$$

The direct consequence of this is the explicit formula for the second Piola–Kirchhoff stress tensor:

(6) $$\tilde{T}=\sum_{\alpha =1}^{N}\omega_{\alpha }\frac{\sigma_{\alpha }}{\lambda_{\alpha }^{2}}\left(e_{\alpha }⊗e_{\alpha }\right)+\tilde{p}C^{-1}.$$

Here, $\tilde{p}\in R$ is an arbitrary number. Mathematically, the term $\tilde{p}C^{-1}$ appears on the right-hand side due to the incompressibility constraint $\dot{C}:C^{-1}=0$. From the mechanical standpoint, the term $\tilde{p}C^{-1}$ is needed since, in incompressible materials, the constitutive law determines the stresses uniquely up to an indefinite hydrostatic part [25].

We call the stress calculation rule (6) the *C-approach* since the fiber stretch $\lambda_{\alpha }$ is defined by Equation (1) using the tensor C. An alternative to this method was proposed in [7] for initially isotropic materials. The concept of representative directions offers various opportunities for an accurate description of the actual mechanical behavior, while preserving the basic principles of constitutive mechanics like objectivity and thermodynamic consistency.

Different versions of the C-approach (6) are possible depending on the specific choice of the representative directions $e_{\alpha }$ and the weighting coefficients $\omega_{\alpha }$. These modifications will be discussed in the following.

### 2.2. Orientation Probability Density Function

Let $S^{2}=\{x\in R^{3}:\;∥x∥=1\}$ be the unit sphere. Each point on $S^{2}$ is identical to a certain fiber orientation vector. Thus, there is a correspondence between the unit sphere and the space of all possible orientations. In this paper, a crucial role is played by the so-called orientation probability density function (OPDF), also known as orientation density function [26] or probability density function [14]. The orientation probability density function f(x) is defined on $S^{2}$:

(7) $$f\left(x\right)⩾0\;\mathrm{for}\;\mathrm{all}\;x\in S^{2}.$$

The OPDF f(x) is assumed continuous on $S^{2}$ and its normalization condition reads $\int_{S^{2}}f\left(x\right)dS=4\pi$, where 4π is the area of the unit sphere. Since each direction is uniquely determined by a couple of opposite points on the unit sphere, we deal with symmetric OPDFs only: f(x)=f(−x) for all $x\in S^{2}$.

Consider a large discrete set of points on the unit sphere. Strictly speaking, we consider a limiting case as the number of points tends to infinity. We say that this set matches the OPDF f(x) if the probability that an arbitrary point is located within the small area dS near $x\in S^{2}$ equals $\frac{f(x)dS}{4\pi }$. This definition is given in the probabilistic setting since there is no need to specify exact location of each individual point on the unit sphere. The special case of the homogeneous (isotropic) OPDF corresponds to f(x)=1 for all $x\in S^{2}$. In this case, all directions are equally important.

Dealing with fibrous composites, the OPDF f(x) tells us how many fibers are oriented along $x\in S^{2}$ in the undeformed state. To simulate an anisotropic mechanical behavior of actual materials, a set of representative directions $\left\{e_{\alpha },\alpha =1,\ldots ,N\right\}$ matching the OPDF f(x) will be needed. In the following, we discuss three different procedures yielding such a set of directions.

### 2.3. Generation of Fiber Sets Matching the Given OPDF

#### 2.3.1. Energy Minimization Method (Landau–Ginzburg Method)

In this subsection we consider a modified version of the algorithm from [27], generating a required heterogeneous set of representative directions (fibers). Within the approach, an initial set of *N* “primary charges” is randomly placed on the unit sphere $S^{2}$. Each “primary charge” gives rise to a “secondary charge” located on the opposite side; secondary charges are attached to the corresponding primary charges. In total, there is a system of 2N charges on the unit sphere. In a spherical coordinate system, the position of the *i*th primary charge is given by its coordinates $\theta_{i},\phi_{i}$ for i=1,…,N. Then, the set of all primary charges is uniquely described by the vector $\vec{q}=\left\{\theta_{1},\phi_{1},\theta_{2},\phi_{2},\ldots ,\theta_{N},\phi_{N}\right\}\in R^{2N}$. Let us consider the total potential energy of the system

(8) $$\Psi \left(\vec{q}\right)=\Psi \left(q_{1},q_{2},\ldots ,q_{2N}\right)=\underset{i<j}{\sum_{i,j=1}^{N}}{\overset{\mathrm{pp}}{\Psi }}_{ij}+{\overset{\mathrm{ss}}{\Psi }}_{ij}+{\overset{\mathrm{ps}}{\Psi }}_{ij}+{\overset{\mathrm{sp}}{\Psi }}_{ij}.$$

Here, ${\overset{\mathrm{pp}}{\Psi }}_{ij}$ is the energy of interaction between two primary charges with numbers *i* and *j*, ${\overset{\mathrm{ss}}{\Psi }}_{ij}$ corresponds to interaction between two secondary charges with numbers *i* and *j*, ${\overset{\mathrm{ps}}{\Psi }}_{ij}$ stands for interaction between the *i*th primary charge and the *j*th secondary charge, ${\overset{\mathrm{sp}}{\Psi }}_{ij}$ corresponds to interaction between the *i*th secondary charge and *j*th primary charge. The Landau–Ginzburg evolution equation is used to minimize the overall energy Ψ:

(9) $$\dot{q_{i}}=-\frac{1}{\eta_{i}}\frac{\partial \Psi }{\partial q_{i}}\;\mathrm{for}\;\mathrm{all}\;i=1,2,\ldots ,N,$$

where $\eta_{i}$ is a pseudo-viscosity; in this study we set $\eta_{i}=1$ for simplicity.

If a homogeneous distribution of representative directions $e_{\alpha }$ (α=1,…,N) is needed, we make use of the following isotropic interaction potential:

(10) $$\Psi_{ij}=\Psi_{ij}\left(r_{ij}\right)=\frac{1}{r_{ij}^{2}},$$

where $r_{ij}=∥x_{i}-x_{j}∥$ is the distance between the involved charges with position vectors $x_{i}$ and $x_{j}$. The minimum of the potential energy Ψ corresponds to an almost homogeneous distribution of representative directions. A similar procedure was already implemented by other authors (see [3]).

**Remark.** Please note that the “charges” are moving on the unit sphere, not in the actual physical space. The physical analogy is used merely as a guideline while constructing the method.

**Remark.** Besides the ansatz (10), there are other potentials which can be used to generate an isotropic set of fibers. For instance, a large set of potentials are implemented within the analysis of nanoscale objects’ self-assembly [28]. However, we prefer working with the power-law potential (10) since it yields a system with a moderate nonlinearity. This property is beneficial for the convergence of numerical procedures.

To generate a set of representative directions matching the given OPDF f(x), the following new potential energy is chosen in the current study:

(11) $$\Psi_{ij}=\frac{1}{r_{ij}^{2}}\frac{1}{\sqrt{f\left(x_{i}\right)f\left(x_{j}\right)}}.$$

Note that the introduced multiplier $1/\sqrt{f\left(x_{i}\right)f\left(x_{j}\right)}$ accounts for the specific OPDF f(x). The impact of this modification will be evident later on. Despite its simplicity, the energy minimization method from this subsection violates Newton’s third law. This can be seen by checking that $\partial \psi_{ij}/\partial x_{i}\neq -\partial \psi_{ij}/\partial x_{j}$. In the next subsection, we suggest another method which is free from this drawback.

#### 2.3.2. Equlibration Method

Similar to the previous subsection, the idea behind the method is to implement a physics-motivated principle, enabling a self-assembly of the required set $\left\{e_{\alpha }\right\}$. Again, *N* primary charges with position vectors $x_{i}$, i=1,2,…,N and *N* opposite secondary charges are placed on the unit sphere. Thus, a total number of 2N charges is considered. We introduce the following ansatz for the repulsive force between two charges located at $x_{i}$ and $x_{j}$:

(12) $$\mathrm{Repulsive}\;{\mathrm{force}}_{ij}=\frac{1}{r_{ij}^{k-1}}\frac{1}{f(x_{i})}\frac{1}{f(x_{j})},\;\mathrm{where}\;r_{ij}=∥x_{i}-x_{j}∥,\;1\leq i\neq j\leq 2N.$$

Various values of *k* are possible; in this work we use k=6.

Consider two charges with the numbers i≠j (1≤i,j≤2N). The repulsive force exerted by the charge *i* on the charge *j* is denoted as $F_{ij}$, and the force exerted by the *j*th charge on the *i*th charge as $F_{ji}$. According to Newton’s third law $F_{ij}=-F_{ji}$. Following (12), we have

(13) $$F_{ij}=-\frac{r_{ij}}{r_{ij}^{k}}\frac{1}{f\left(x_{i}\right)f\left(x_{i}\right)},\;r_{ij}=x_{i}-x_{j}.$$

Let there be an array [X] of position vectors of charges. Since the position of the secondary charges depends on the primary charges, it is sufficient to consider only the primary charges:

(14) $$X={\left(x_{1},x_{2},\ldots ,x_{N}\right)}^{T}.$$

For them, we define the velocities and accelerations

(15) $$\dot{X}={\left({\dot{x}}_{1},{\dot{x}}_{2},\ldots ,{\dot{x}}_{N}\right)}^{T},\;\ddot{X}={\left({\ddot{x}}_{1},{\ddot{x}}_{2},\ldots ,{\ddot{x}}_{N}\right)}^{T}.$$

Here, the superimposed dot stands for the time derivative. Since each charge moves along the unit sphere, we have ${\dot{x}}_{i}\cdot x_{i}=0$ for i=1,2,…,N. Let the array of tangent forces be represented as follows:

(16) $$F^{\tan\mathrm{gent}}={\left(F_{1}^{\tan\mathrm{gent}},F_{2}^{\tan\mathrm{gent}},\ldots ,F_{N}^{\tan\mathrm{gent}}\right)}^{T},\;F_{i}^{\tan\mathrm{gent}}=\left(1-x_{i}⊗x_{i}\right)F_{i},$$

where $\left(1-x_{i}⊗x_{i}\right)$ is the projection operator and $F_{i}$ is the total force exerted on the primary charge *i* by other charges:

(17) $$F_{i}=\underset{j\neq i}{\sum_{j=1}^{N}}{\overset{p}{F}}_{ji}+{\overset{s}{F}}_{ji}\;\mathrm{for}\;\mathrm{all}\;i=1,2,\ldots ,N.$$

Here, ${\overset{p}{F}}_{ji}$ is the force exerted on the *i*th primary charge by the *j*th primary charge; ${\overset{s}{F}}_{ji}$ is exerted by the *j*th secondary charge. The force exerted by the secondary charge *i* on the primary charge *i* is neglected, since it acts exactly along the normal to the sphere.

Using Newton’s second law, we obtain the equations of motion for the set of primary charges:

(18) $$m\ddot{X}=F^{\tan\mathrm{gent}},$$

where *m* is a pseudo-mass; we use m=1 in this work. This differential equation accounts for the interactions between the charges, but there is no viscosity. To enable equilibration of charges due to viscous dissipation, the following modification is considered:

(19) $$m\ddot{X}=F^{\tan\mathrm{gent}}-c\dot{X}.$$

Here, c>0 is the pseudo-viscosity. Due to energy dissipation, $\dot{X}\to 0$ and $\ddot{X}\to 0$ as t→∞. Thus, $F^{\tan\mathrm{gent}}\to 0$ as t→∞, which means that the system of charges comes to an equilibrium. The numerical implementation of this method is described in Appendix A.

**Remark.** In general, there is a minor probability of the overall system of charges getting stuck in a meta-stable state far from the optimal equilibrium. To avoid this undesired effect, a random force may be added to the right-hand side of the motion Equation (19). This idea is already used in some molecular dynamics simulations on a sphere [28].

**Remark.** Both methods—the energy minimization method and the method of equilibration — are insensitive to the scaling of the OPDF f(x). In other words, the normalization condition $\int_{S^{2}}f\left(x\right)dS=4\pi$ does not have to be satisfied. In contrast to the method from the previous subsection, the equilibration method respects Newton’s third law.

**Remark.** The methods from Section 2.3.1 and Section 2.3.2 are inspired by physical analogies involving quasi-elastic (conservative) interactions between charges on the unit sphere. An alternative method can be designed starting from the general mechanical principle of entropic ordering, see [29] for the background information.

#### 2.3.3. Voronoi Cells Method

The third method generating a set of fibers is based on the Voronoi tessellation [30]. In contrast to the well-known Voronoi cells in $R^{n}$, a tasselation on the unit sphere is considered here. The idea behind the algorithm is to establish a relation between individual fibers and Voronoi cells on $S^{2}$. This method is purely mathematical without any physical motivation behind it. Assume that we need to generate *N* fibers matching the given OPDF f(x). Such a set corresponds to 2N Voronoi cells. The corresponding Voronoi tessellation is uniquely determined by setting 2N sites (also known as generators) on $S^{2}$. We denote these sites as $x_{1},x_{2},...,x_{2N}$. To reduce the number of degrees of freedom, we assume that the position vectors of only the first *N* sites are independent:

(20) $$x_{N+1}=-x_{1},\;x_{N+2}=-x_{2},\ldots ,\;x_{2N}=-x_{N}.$$

Next, let $z_{i}$ be the center of mass of the *i*th Voronoi cell. Obviously, the set $\left\{z_{1},z_{2},\ldots ,z_{N}\right\}$ is uniquely determined by the set $\left\{x_{1},x_{2},\ldots ,x_{N}\right\}$. Within our method the required set of *N* fibers is as follows: $e_{1}=z_{1}/∥z_{1}∥$, $e_{2}=z_{2}/∥z_{2}∥$, …, $e_{N}=z_{N}/∥z_{N}∥$.

Recall that 4π is the area of the unit sphere; let $S_{i}$ be the area of the *i*th cell. To find the desired heterogeneous fiber distribution, the following objective function is minimized:

(21) $$\Phi_{\mathrm{Voronoi}}\left(x_{1},x_{2},\ldots ,x_{2N}\right)=\sum_{i=1}^{N}{\left(S_{i}-\frac{4\pi }{2Nf(e_{i})}\right)}^{2}.$$

The perfect situation $\Phi_{\mathrm{Voronoi}}=0$ means that the area of the *i*th Voronoi cell is inversely proportional to the OPDF f(x) evaluated at the center of the cell:

(22) $$S_{i}=\frac{4\pi }{2Nf(e_{i})}.$$

To check that the Voronoi cells method is assymptotically exact, let us consider an arbitrary $x\in S^{2}$ and a small surface element dS near x. According to (22), the area of the Voronoi cells near x behaves like $\frac{4\pi }{2Nf(x)}$ as N→∞. Thus, the surface element dS contains $f\left(x\right)dS\frac{2N}{4\pi }$ Voronoi cells. Since there are 2N cells, the probability that an arbitrary cell is contained within dS equals $f\left(x\right)\frac{dS}{4\pi }$. This estimate means that the generated set $\left\{e_{\alpha }\right\}$ matches the given OPDF.

There is also an alternative way to build the error functional:

(23) $$\Phi_{\mathrm{Voronoi}}\left(x_{1},x_{2},\ldots ,x_{2N}\right)=\sum_{i=1}^{N}{\left(S_{i}f\left(e_{i}\right)-4\pi /\left(2N\right)\right)}^{2}.$$

This version of the error functional is beneficial when a division by zero is possible in (21), that is, when f=0 in a certain area. In the simplest case of the uniform distribution (f=1), the absolute minimum of the error function corresponds to the following set of equations: $S_{i}=4\pi /\left(2N\right)$, i=1,…,N.

Please note that the error functions (21) and (23) can only be used for OPDFs satisfying the normalization condition: $\int_{S^{2}}f\left(\vec{x}\right)dS=4\pi$. However, if the OPDF f(x) for some reason does not obey the normalization condition, then another pair of error functions should be employed:

(24) $$\Phi_{\mathrm{Voronoi}}\left(x_{1},x_{2},\ldots ,x_{2N}\right)=\sum_{i=1}^{N}{\left(S_{i}-\frac{MULT\cdot 4\pi }{2Nf(e_{i})}\right)}^{2}.$$

(25) $$\Phi_{\mathrm{Voronoi}}\left(x_{1},x_{2},\ldots ,x_{2N}\right)=\sum_{i=1}^{N}{\left(\frac{f(e_{i})}{MULT}S_{i}-4\pi /\left(2N\right)\right)}^{2}.$$

Here, $MULT=\sum_{i=1}^{N}\frac{2S_{i}\cdot f\left(e_{i}\right)}{4\pi }$ is a correction multiplier that allows us to deal with various OPDFs, normalized and non-normalized.

For the numerical implementation of the algorithm, the unit sphere is uniformly covered with a large number of so-called control points. A computationally efficient way to generate such a set of control points is explained in Appendix B. Let $N_{\mathrm{control}}$ be the number of these points, $N_{\mathrm{control}}\gg N$. For each control point $y_{k}$, $k\in \{1,\ldots ,N_{\mathrm{control}}\}$, we find the site on the unit sphere closest to this control point:

(26) $$j=\underset{l=1,\ldots ,2N}{\mathrm{argmin}}∥y_{k}-x_{l}∥.$$

Per definition, the proximity to the site $x_{j}$ means that the control point $y_{k}$ falls into the *j*th Voronoi cell. Let $M_{i}$ be the total number of control points that fell into the *i*th Voronoi cell (i=1,2,…,2N). Then, the area of the *i*th Voronoi cell is calculated by the approximate formula:

(27) $$S_{i}=\frac{M_{i}}{N_{\mathrm{control}}}\cdot 4\pi ,\;i=1,2,\ldots ,2N.$$

**Figure 1:**
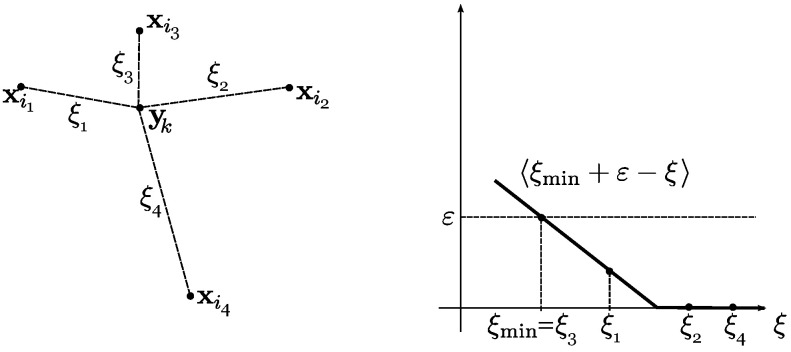
Voronoi cell smoothing method. Left: control point $y_{k}$ is claimed by four sites $x_{i_{1}},x_{i_{2}},x_{i_{3}},x_{i_{4}}$ from its neighbourhood. Right: dependence of the primary weighting coefficient $\omega_{j}$ related to the site $x_{i_{j}}$ on the distance to the control point $y_{k}$.

The disadvantage of using Equation (27) is that the areas $S_{i}$ are discontinuous functions of the position vectors of the sites $x_{1},x_{2},\ldots ,x_{2N}$. The following modification of the algorithm consists in “smearing” the Voronoi cells. In the case of smearing, a situation may arise when several Voronoi cells claim certain control points. The following steps are taken to resolve the conflict. For a given $k\in \{1,2,\ldots ,N_{\mathrm{control}}\}$ assume that $x_{i_{1}},x_{i_{2}},x_{i_{3}},x_{i_{4}}$ are the sites on the unit sphere corresponding to the smallest $\xi_{j}=∥y_{k}-x_{i_{j}}∥$ ($1\leq i_{1},i_{2},i_{3},i_{4}\leq 2N$), see Figure 1 (left). Here we consider only four sites closest to the control point $y_{k}$; the situation where five or more Voronoi cells claim one control point is ignored as quite rare. The areas of the cells are calculated using the following algorithm:

Step 1: For a given $k\in \{1,\ldots ,N_{\mathrm{control}}\}$ compute the primary weight of the site $x_{i_{j}}$, where j=1,2,3,4:

(28) $$\omega_{j}=\langle \xi_{\min}+\varepsilon -\xi_{j}\rangle .$$

Here, $\xi_{\min}$ is the smallest value of $\xi_{j}=∥y_{k}-x_{i_{j}}∥$, j=1,2,3,4; ε>0 is a smoothing parameter; 〈x〉=max(x,0) is the Macaulay bracket, see Figure 1 (right).

Step 2: Find the final weight of the site $x_{i_{j}}$:

(29) $$W_{j}=\frac{\omega_{j}}{\omega_{1}+\omega_{2}+\omega_{3}+\omega_{4}},\;j=1,2,3,4.$$

Within a loop over all control points, each control point $y_{k}$ contributes its parts $W_{1}$, $W_{2}$, $W_{3}$, and $W_{4}$ to the cells with the numbers $i_{1}$, $i_{2}$, $i_{3}$, and $i_{4}$:

(30) $$S_{i_{j}}\leftarrow S_{i_{j}}+W_{j}\cdot 4\pi \;\mathrm{for}\;\mathrm{all}\;j=1,2,3,4.$$

Here, the symbol A←B means assigning the value *B* to the variable *A*.

Owing to the smoothing, we ensure a continuous dependence of the cell areas $S_{1}$, …, $S_{N}$ on $x_{1}$, $x_{2}$, …, $x_{N}$. The center of mass of the *i*th cell is defined as the center of mass of the set of control points that fall into this cell. In this case, each control point is included with a weighting coefficient calculated by Equation (29). The error functionals (21)–(25) are minimized by the Levenberg–Marquardt method [31,32].

#### 2.3.4. Demonstration Tests: Generated Sets of Fibers

The energy minimization method, the equilibration method, and the Voronoi cells method are tested in this subsection. Towards that end, three different types of the OPDF are used.

**Figure 2:**
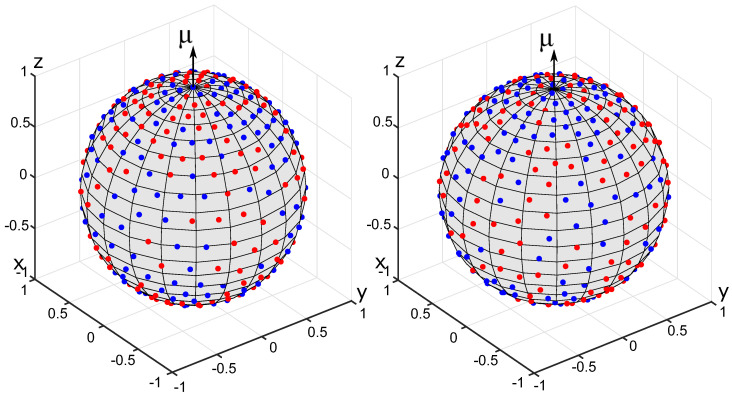
Anisotropic sets of 200 fibers generated for the von Mises-Fischer OPDF. The fibers are represented by charges on the unit sphere. The primary and secondary charges are shown in blue and red, respectively. **Left**: energy minimization method. **Right**: equilibration method.

**Figure 3:**
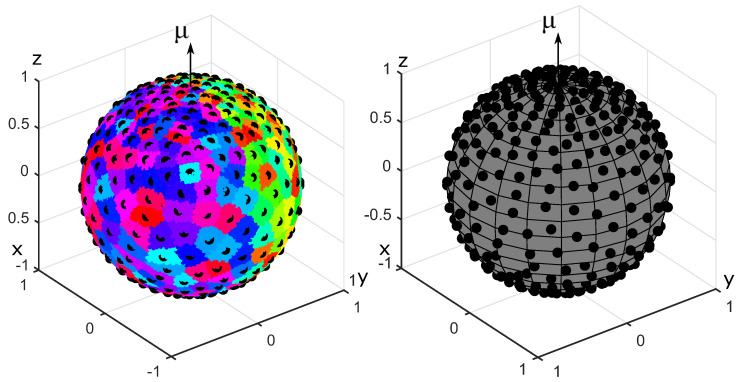
Results of generating anisotropic fiber systems using the Voronoi cells method for the von Mises-Fischer OPDF with N=200. The centers of mass of Voronoi cells are shown by black dots. **Left**: Voronoi cells. **Right**: Centers of mass of Voronoi cells.

**OPDF of von Mises–Fischer type.** Within the first series of tests, the OPDF of von Mises–Fisher type is considered:

(31) $$f^{\mathrm{MF}}=f^{\mathrm{MF}}\left(x,\mu ,k\right)=C_{3}\left(k\right)\frac{e^{k\mu \cdot x}+e^{-k\mu \cdot x}}{2},$$

where k≥0 is the degree of heterogeneity, μ is the orientation vector for the OPDF (∥μ∥=1), μ·x is the scalar product of μ and x, $C_{3}\left(k\right)=\left(2k\right)/\left(e^{k}-e^{-k}\right)$ is the normalization constant. OPDFs of this type are used, among others, to describe the distribution of collagen fibers in biological soft tissues [22]. For testing purposes, we set k=2, $\mu ={\left(0,0,1\right)}^{T}$. The results of generating a set of N=200 fibers with the energy minimization method and the equilibration method are shown in Figure 2. The results of the Voronoi cells method are shown in Figure 3. Please recall that the generated sets $\left\{e_{\alpha }\right\}$ are represented as collections of points on the unit sphere.

**Figure 4:**
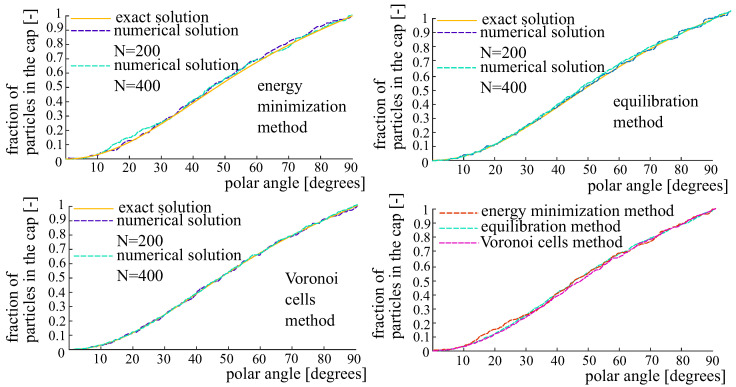
Cumulative distribution functions pertaining to the von Mises–Fischer distribution. **Top left**: the energy minimization method. **Top right**: the equilibration method. **Bottom left**: the Voronoi cells method. **Bottom right**: comparison of the three methods for N=400 fibers.

A spherical dome with the polar angle θ∈[0,π/2] is a set of points on the unit sphere such that the angle between their position vector and the orientation vector μ is less than θ. A cumulative distribution function gives the portion of charges located within the spherical dome as a function of its polar angle. The cumulative distribution function for the von Mises–Fischer OPDF is shown in Figure 4 for all three generation methods.

**OPDF of Valée-Poussin type.** The next test is based on the OPDF of Vallée-Poussin type:

(32) $$f^{\mathrm{VP}}=\frac{1}{8}\frac{\beta (\frac{3}{2},\frac{1}{2})}{\beta (\frac{3}{2},k+\frac{1}{2})}\left(\cos^{2k}\left(\frac{\omega }{2}\right)-\cos^{2k}\left(\frac{\pi -\omega }{2}\right)\right),\;\mathrm{where}\;\omega =\arccos\left(\frac{\mu \cdot x}{∥\mu ∥∥x∥}\right).$$

**Figure 5:**
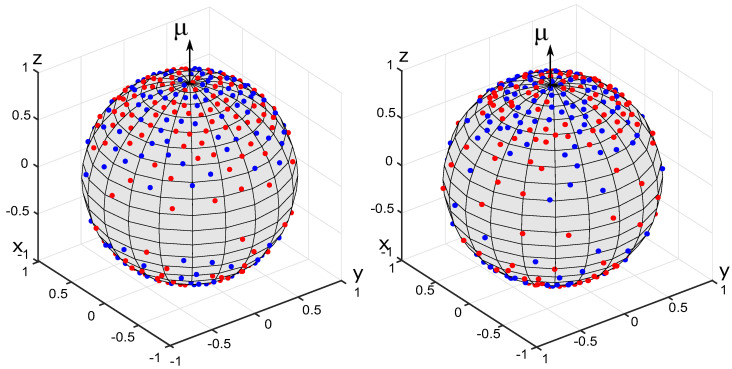
Generated systems of 200 fibers pertaining to the OPDF of Vallée-Poussin type. The fibers are represented by charges on the unit sphere. The primary and secondary charges are shown in blue and red. **Left**: Energy minimization method. **Right**: The method of equilibration.

**Figure 6:**
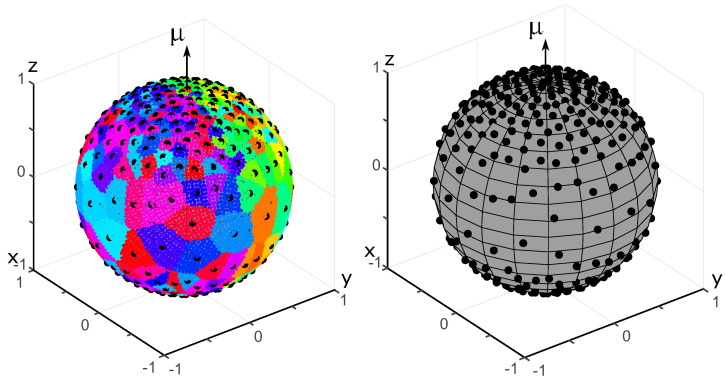
Generated 200 fibers using the Voronoi cells method for the OPDF of Vallée-Poussin type. **Left**: Voronoi cells. **Right**: Centers of mass of the Voronoi cells.

**Figure 7:**
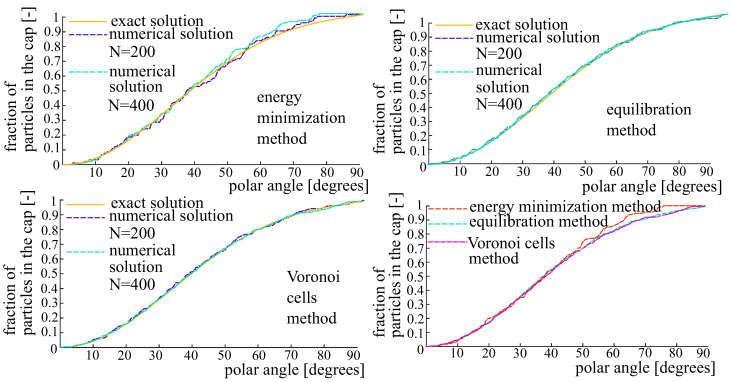
Cumulative distribution function for the OPDF of Vallée-Poussin type. **Top left**: energy minimization method. **Top right**: the equilibration method. **Bottom left**: the Voronoi cells method. **Bottom right**: comparison of the three methods for N=400 fibers.

Here, β(·,·) is the Euler Beta-function; μ is the orientation vector. To be definite, we set k=5 and $\mu ={\left(0,0,1\right)}^{T}$. Figure 5 shows the generated sets of N=200 fibers using the energy-minimization method and the equilibration method. Figure 6 corresponds to the Voronoi cells method. The cumulative distribution functions are shown in Figure 7.

**OPDF as a quadratic form.** As a third test, we consider the following probability density function given by the quadratic form

(33) $$f^{\mathrm{quadratic}}\left(x\right)=x\cdot M\cdot x,$$

where M is a symmetric, positive definite tensor. In the context of material modeling, such a tensor can be seen as a structure tensor, governing orthotropic material properties. To be definite, we use (up to a positive normalization multiplier):

(34) $$M=\left(\begin{array}{ccc} 1 & 0 & 0\\ 0 & 4 & 0\\ 0 & 0 & 3 \end{array}\right).$$

**Figure 8:**
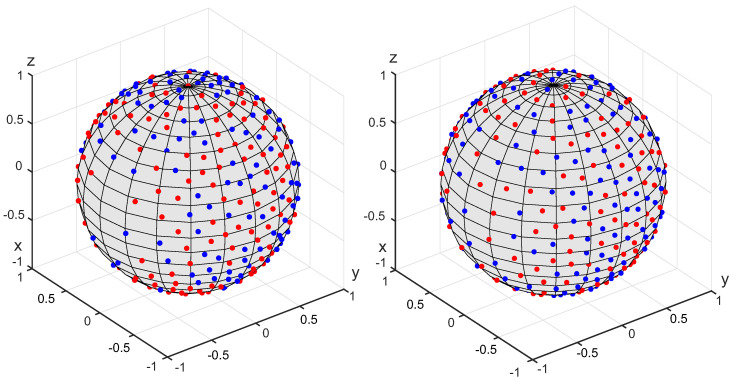
Generated sets of 200 fibers pertaining to the OPDF as a quadratic form. The fibers are represented by charges on the unit sphere. The primary and secondary charges are shown in blue and red. **Left**: Energy minimization method. **Right**: The method of equilibration.

**Figure 9:**
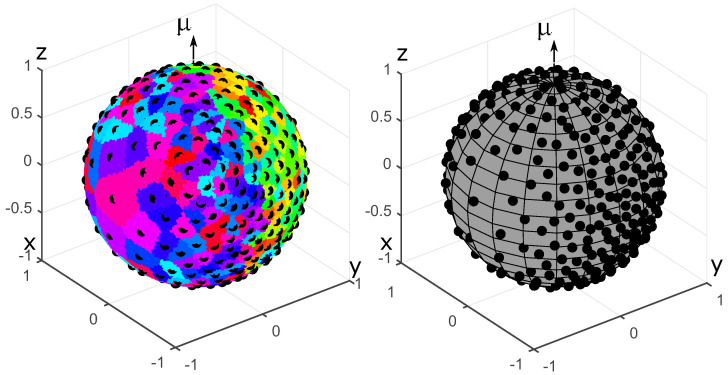
Voronoi cells method used to generate 200 fibers. The OPDF is given by the quadratic form. **Left**: the Voronoi cells. **Right**: centers of mass of the Voronoi cells.

**Figure 10:**
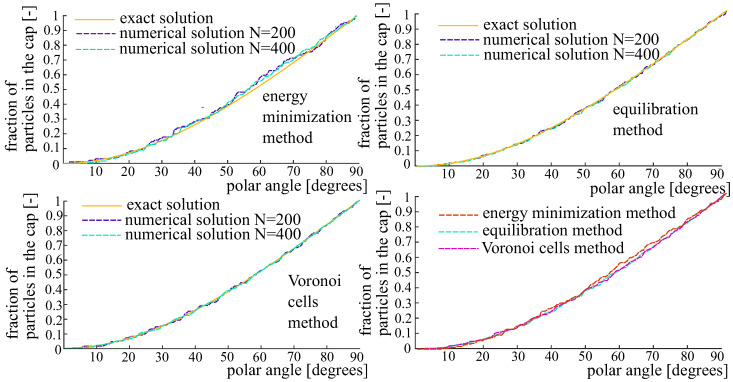
Cumulative distribution functions for the OPDF given by the quadratic form. **Top left**: the energy minimization method. **Top right**: the uquilibration method. **Bottom left**: the Voronoi cells method. **Bottom right**: comparison of the three methods for N=400 fibers.

Figure 8 shows the generated set of N=200 fibers using the energy minimization and the equilibration methods; Figure 9 corresponds to the Voronoi cells method. Unlike the two previous examples, the OPDF is not transversally isotropic, namely, the distribution is orthotropic. The cumulative distribution functions pertaining to this OPDF are shown in Figure 10.

The key assumption behind the energy minimization method is ansatz (11) describing the potential energy function. According to (11), $\psi ∼\frac{1}{\sqrt{f_{i}f_{j}}}$. However, within the equilibration method Equation (12) gives the repulsion force between two charges in the form: $\mathrm{Force}∼\frac{1}{f_{i}f_{j}}$. These two assumptions are based on trial-and-error without a proper mathematical justification. Numerical tests show that both approaches yield reasonable results, both in transversely isotropic and orthotropic cases.

According to Equations (21) and (23), the Voronoi cells method is asymptotically exact: it allows obtaining the desired distribution as N→∞. As revealed by the numerical tests, the most accurate results are provided by the equlibration method and the Voronoi cells method. In particular, the superior accuracy of these two methods is visible from the cumulative distributions (Figure 4, Figure 7 and Figure 10). If needed, a strict comparison of the three generation methods as well as rigorous convergence studies can be carried out in terms of a newly proposed mechanics-based metric [33].

### 2.4. Two Specific Versions of the Concept of Representative Directions

Two different versions of the C-approach (6) will be considered in this section to model initially anisotropic materials. The first version is called “anisotropy stored in weights”. It is based on the isotropic distribution of the representative directions where the entire anisotropy is contained in the weighting factors $\omega_{\alpha }=f\left(e_{\alpha }\right)/N$:

(35) $$\tilde{T}=\frac{1}{N}\sum_{\alpha =1}^{N}f\left(e_{\alpha }\right)\frac{\sigma_{\alpha }}{\lambda_{\alpha }^{2}}\left(e_{\alpha }⊗e_{\alpha }\right)+\tilde{p}C^{-1},\;\mathrm{directions}\;\mathrm{distributed}\;\mathrm{homogeneously}.$$

The second version of the C-approach is called “anisotropy stored in directions”. It implements the anisotropic set of fibers, related to the OPDF f(x) and constant weighting coefficients $\omega_{\alpha }=1/N$:

(36) $$\tilde{T}=\frac{1}{N}\sum_{\alpha =1}^{N}\frac{\sigma_{\alpha }}{\lambda_{\alpha }^{2}}\left(e_{\alpha }⊗e_{\alpha }\right)+\tilde{p}C^{-1},\;\mathrm{directions}\;\mathrm{match}\;\mathrm{the}\;\mathrm{OPDF}\;f\left(x\right).$$

**The main idea.** Note that the first version treats all regions on the unit sphere in the same way, even those regions where the OPDF is exactly zero. In that sense, a big amount of computations are useless since corresponding stresses $\sigma_{\alpha }$ are multiplied with $f(e_{\alpha })\approx 0$. In contrast to the first version, the second version treats the regions on the unit sphere with a large value of the OPDF more accurately than the regions where the OPDF is small. Due to the automatic adjustment of directions, a more reasonable use of computational power is ensured. Therefore, for strongly anisotropic OPDFs, the second version, called “anisotropy stored in directions”, *is computationally more efficient and reasonable*.A practically important case when the OPDF is not known a-priori will also be considered.

**Remark.** Researchers dealing with the concept of representative directions (or similar approaches) have recognized the urgent need to reduce the computational costs. In [13,14] the authors utilized the mechanical effect that zero stresses can be expected in fibers under compression; the required computational speed-up was achieved by excluding fibers with zero stresses. However, the approach proposed in the current study is based on the different idea of using a higher resolution in the domain of large OPDF.

## 3. Actual Anisotropic Material

### 3.1. Polymeric Fibrous Material Produced by Electrospinning

Electrospinning (ES) is an innovative technology of non-woven fibrous materials production from a solution of polymers, both synthetic and natural [34,35]. Since ES enables fiber production from mixtures of polymers and blends with drugs or biologically active substances [36], ES is widely used in tissue engineering. In particular, regenerative medicine employs ES-produced vascular grafts (VG), mimicking actual biological tissues [35,37,38,39].

Varying the ES conditions like the used electrode (RF patent N2704314), rotation speed of the collector, and velocity of jet motion [35,39], one can produce VGs with highly anisotropic mechanical properties [40]. The accurate programming of deformation patterns of VGs is crucial for compliance with natural arteries by correct anastomosis, which is necessary for long-term functioning of VGs [41,42]. In this paper, we focus on the description of electrospun poly-(butylene terephthalate) [23]. Other anisotropic electrospun-produced matrices can be dealt with in a similar way.

### 3.2. Uniaxial Material Law and Orthotropic OPDF

To describe the mechanical behavior of a single representative direction (fiber), the following one-dimensional material law is utilized:

(37) $$\begin{array}{c} \sigma_{\alpha }^{\mathrm{eng}}=0\;\mathrm{for}\;\varepsilon_{\alpha }^{\mathrm{eng}}\leq 0,\\ \sigma_{\alpha }^{\mathrm{eng}}=\min\left(a_{1}\varepsilon_{\alpha }^{\mathrm{eng}},a_{1}\varepsilon_{0}+a_{2}\left(\varepsilon_{\alpha }^{\mathrm{eng}}-\varepsilon_{0}\right)\right)\;\mathrm{for}\;\varepsilon_{\alpha }^{\mathrm{eng}}>0. \end{array}$$

**Figure 11:**
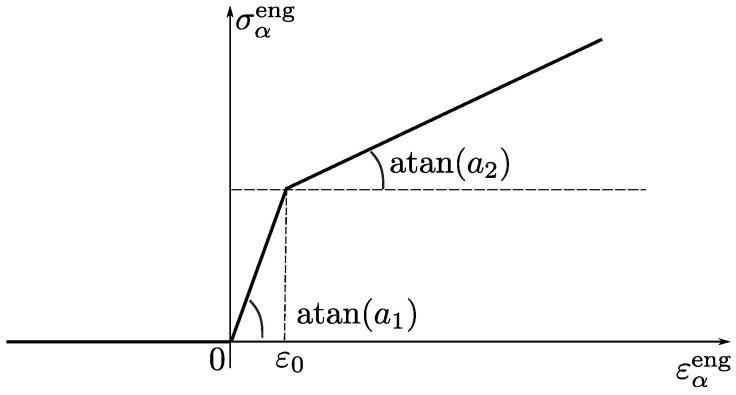
One-dimensional material law implemented for each representative direction.

Here, engineering stresses and strains are used. This non-linear elastic mechanical behavior of a single fiber is sketched in Figure 11. Since the standard C-approach operates with true stresses (see Equation (6)), the following geometric pre- and post-processing steps are needed for each direction: $\varepsilon_{\alpha }^{\mathrm{eng}}:=\lambda_{\alpha }-1$ (pre-processor), $\sigma_{\alpha }^{\mathrm{true}}:=\sigma_{\alpha }^{\mathrm{eng}}\left(1+\varepsilon_{\alpha }^{\mathrm{eng}}\right)$ (post-processor).

Aiming at the simulation of polymeric materials produced by electrospinning, the following orthotropic OPDF will be implemented:

(38) $$f\left(x\right)={\left(x\cdot M\cdot x\right)}^{m}.$$

Here, M is a symmetric, positive definite structural tensor and m>0 is a material parameter. In the axes of orthotropy $k_{1}$, $k_{2}$, $k_{3}$ the structural tensor M takes the following form:

(39) $$M=m_{1}k_{1}⊗k_{1}+m_{2}k_{2}⊗k_{2}+m_{3}k_{3}⊗k_{3}.$$

The vectors $k_{1}$, $k_{2}$, and $k_{3}$ are understood as material directions.

Due to strong anisotropy which appears when $\min\left(m_{1},m_{2},m_{3}\right)\ll \max\left(m_{1},m_{2},m_{3}\right)$, the simulation may become unstable. To stabilize computations, a regularization is carried out by reinforcing the material by an additional neo-Hookean material:

(40) $${\tilde{T}}_{\mathrm{reg}}=\beta {\bar{C}}^{D}\cdot C^{-1},$$

where $\bar{C}=\det{\left(C\right)}^{-1/3}C$ is the isochoric part of the right Cauchy–Green tensor and β>0 is a small regularization parameter.

### 3.3. Calibration of the Material Model against Experimental Data

**Figure 12:**
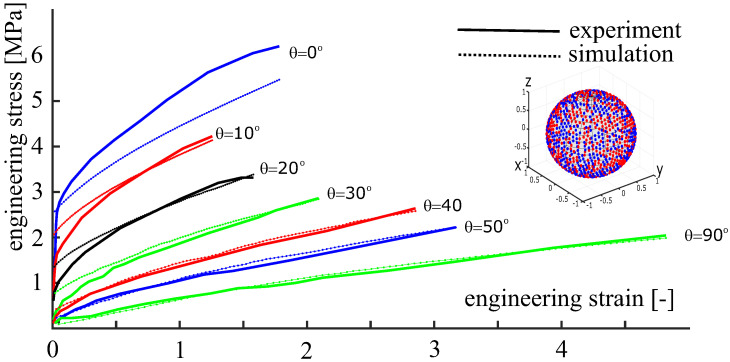
Experimental data from [23] and simulation results obtained by the first version of the approach (anisotropy stored in weights) with N=800 uniformly distributed fibers. In each test, the tensile axis is inclined at the angle θ to the hoop direction.

To demonstrate the applicability of the approach to initially anisotropic materials, the mechanical behavior of a polymeric material produced by electrospinning is analyzed. The calibration of the anisotropic material model is carried out against actual experimental data from [23]. The set of experimental data is formed by a series of uniaxial tension tests. To describe these tests, let θ be the angle between the sample’s axis and the hoop direction of the ES-collector. Within the experimental program, the angle θ ranges from $0^{\circ }$ (tension along the hoop direction) to $90^{\circ }$ (tension along the axial direction). As is seen from Figure 12, the material exhibits a very strong anisotropy. The samples oriented along the collector’s hoop direction exhibit the largest stiffness. This is due to the fibers of the ES-produced material being mostly oriented along the hoop direction.

The homogeneous stretching of a sample cut along an arbitrary material direction k is modeled in the following way. First, the rotation of the sample is simulated so that the material direction k becomes oriented along $e_{1}$. Second, the stretching of the sample along $e_{1}$ is modeled.

Let $Q=\exp(\theta \varepsilon e_{3})$ be the rotation tensor, where exp(·) is the tensor exponential; θ is the angle of rotation, ε is the permutation tensor (Levi–Chivita tensor), and $e_{3}$ is the axis of rotation. From the condition $Qk=e_{1}$ it follows that the deformation of the sample is described as follows:

(41) $$F\left(t\right)=F^{\left(e_{1}\right)}\left(t\right)\cdot Q,\;F^{\left(e_{1}\right)}\left(t\right)=\left(\begin{array}{ccc} F_{11}\left(t\right) & F_{12}\left(t\right) & F_{13}\left(t\right)\\ 0 & F_{22}\left(t\right) & F_{23}\left(t\right)\\ 0 & 0 & F_{33}\left(t\right) \end{array}\right).$$

Here, the operator $F^{\left(e_{1}\right)}\left(t\right)$ describes the homogeneous deformation of the sample during the stretching phase. The coordinate $F_{11}\left(t\right)$ is explicitly defined through the prescribed engineering strain ε: $F_{11}\left(t\right)=1+\varepsilon \left(t\right)$. Due to material’s incompressibility we have $F_{33}=1/\left(F_{11}F_{22}\right)$. The remaining four unknown coordinates of F are determined through the system of four scalar equations:

(42) $$\left(T_{12},T_{13},T_{23},T_{22}-T_{33}\right)=\left(0,0,0,0\right).$$

Here, $T_{ij}$ are the coordinates of the Cauchy stress tensor. The system of four nonlinear Equation (42) is solved by the Levenberg–Marquardt method at each load increment (each time step). After finding all the coordinates of the deformation gradient tensor F and the Cauchy stress tensor T, we correct the hydrostatic part:

(43) $$T^{\mathrm{correct}}=T-T_{22}\cdot 1.$$

This correction yields the desired uniaxial stress state such that $T_{22}^{\mathrm{correct}}=T_{33}^{\mathrm{correct}}=0$.

To enable simulations, the parameters $a_{1}>0$ and $a_{2}>0$ of the one-dimensional material model are needed. Additionally, the parameters of the orthotropy $m_{1}>0$ and $m_{2}>0$ as well as the exponent *m* are subject to identification. The remaining parameter $m_{3}>0$ should be determined from the normalization condition $\int_{S^{2}}f\left(\vec{x}\right)dS=S$. However, we implement methods which are stable regarding the violation of the normalization condition. For simplicity of the identification procedure, we fix the remaining parameter: $m_{3}=0.8$.

During the parameter identification stage, we implement the first version of the C-approach given by Equation (35). The advantage of the first version is that for each new OPDF, the same homogeneous set of fibers can be used. To enable a high simulation accuracy, we employ N=800 homogeneously distributed fibers. Note that the computationally more efficient version of the C-approach corresponding to Equation (36) should not be used during model calibration, since it would require a new set of representative directions at each iteration of the calibration procedure.

The small regularization parameter is set to $\beta =\frac{N(a_{1}+a_{2})}{50000}$. Six unknown parameters ($m_{1}$, $m_{2}$, *m*, $\varepsilon_{0}$, $a_{1}$, $a_{2}$) were determined by minimizing an error function using the Nelder–Mead method [43]. The identified parameters are summarized in Table 1. The corresponding simulation results are shown in Figure 12. As is seen from the figure, the C-approach (35) combined with the implemented one-dimensional material law and the chosen OPDF describes the given experimental data with a plausible accuracy. The discrepancy between the theory and experiment at $\theta =0^{\circ }$ is due to the restrictive ansatz (38) used for the OPDF. A relatively low accuracy at small strains is caused by the simplified nature of the one-dimensional material law (37).

**Remark.** As already mentioned, the considered OPDF does not satisfy the normalization condition, that is, $\int_{S^{2}}\frac{f(\vec{x})dS}{S}\neq 1$. When identifying material constants, we neglect the normalization condition to speed up the calculations. At the stage of identification of material constants, the lack of normalization is compensated by the appropriate selection of constants $a_{1}$ and $a_{2}$.

**Remark.** In this section, the unknown parameters of the OPDF f(x) were identified based on the series of tension tests, which provide relevant data on material’s anisotropy. An even bigger body of useful information regarding the anisotropy can be obtained by optical methods like DIC [44].

**Figure 13:**
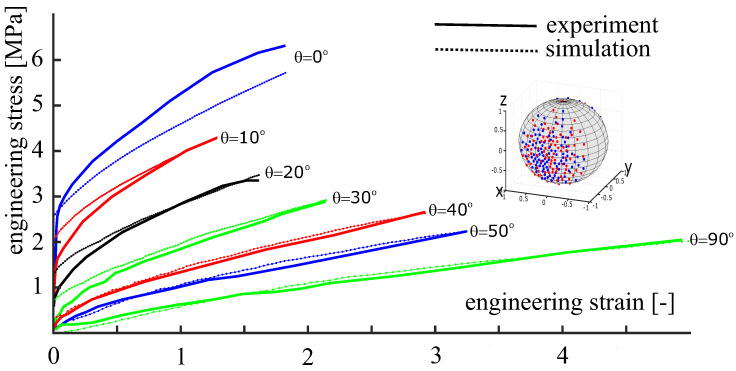
Experimental data from [23] and simulation results obtained by the second version of the approach (anisotropy stored in directions) with N=200 heterogeneously distributed fibers. In each test, the tensile axis is inclined at the angle θ to the hoop direction.

Following the main idea of the current study, we reduce the number of representative directions by switching to the second version of the concept, see Equation (36). Towards that end, an anisotropic set of N=200 fibers is generated, matching the previously identified OPDF. The accurate Voronoi cells method is employed here. The same set of material parameters is used in the simulations (Table 1). The simulation results obtained using 200 heterogeneously distributed fibers are shown in Figure 13. We see that the results based on N=800 homogeneously distributed fibers can be reproduced using the anisotropic set of only N=200 fibers. This reduction of the number of representative directions has a positive effect on the efficiency of the computational procedure.

## 4. Discussion and Conclusions

The well-known concept of representative directions is considered in this study, allowing us to generalize uniaxial material models to multi-axial constitutive relations. The standard C-approach is used here, cf. Equation (6). In the current paper we have shown that this approach can be adjusted to initially anisotropic materials by employing an appropriate OPDF f(x) in two different ways. The first version of the C-approach implements a homogeneous set of representative directions (fibers) and different weighting coefficients, cf. Equation (35). The second version implements a heterogeneous set of fibers with constant weighting coefficients, cf. (36).

The main contribution of the manuscript is that a computationally efficient version of the concept is suggested. The underlying idea is to treat only essential directions where the OPDF is large and neglect directions with a small OPDF. However, the high efficiency comes at a cost of preparing a heterogeneous set of fibers, matching the given OPDF. Although computationally expensive, this preparation step is carried out “offline” before the start of the actual simulation.

Three different algorithms are suggested, each yielding the required heterogeneous set of fibers. These algorithms have been tested using three different OPDFs. In our opinion, the most accurate algorithm is based on the Voronoi method. However, the other two methods can be also used to create a good initial approximation for the Voronoi cells method, thus improving its convergence and robustness.

In this paper, the unknown OPDF f(x) pertaining to the specific material is identified within the model calibration procedure. Since the proper OPDF is not known during the calibration, the first version of the C-approach (anisotropy is stored in the weighting coefficients) should be used, although it is less efficient. After the optimal OPDF providing the best possible fit is found, the corresponding heterogeneous set of fibers is generated for later use in combination with the second version of the C-approach (anisotropy is stored in the fiber distribution). Thus, a two-stage procedure employing both versions of the C-approach is advocated here to model actual materials.

The procedure can be essentially simplified if the OPDF f(x) is known a-priori. For instance, the OPDF can be identified by imaging analysis for some fibrous materials [45]. In that case, the computationally efficient version of the C-approach can be used immediately after the heterogeneous set of fibers is generated.

For testing purposes, a series of tension tests carried out in different directions is taken from [23]. The simulation results exhibit a good correspondence with the available experimental data for the considered ES-produced polymeric material with a pronounced initial anisotropy (Figure 12 and Figure 13). Alternatively, the affine C-approach can be calibrated against synthetic experimental data, provided by advanced non-affine approaches [46,47]. The efficient use of computational power within the second version of the C-approach (anisotropy stored in the fiber distribution) opens up broad prospects for the concept of representative directions regarding a number of practical problems.

## Figures and Tables

**Table 1:** Identified parameters for the structural tensor M and the one-dimensional material law.

$m_{1}$ [-]	$m_{2}$ [-]	*m* [-]	$\varepsilon_{0}$ [-]	$a_{1}$ [MPa]	$a_{2}$ [MPa]
2.89	0.063	2.588	0.0034	423.82	0.52

**Figure A1:**
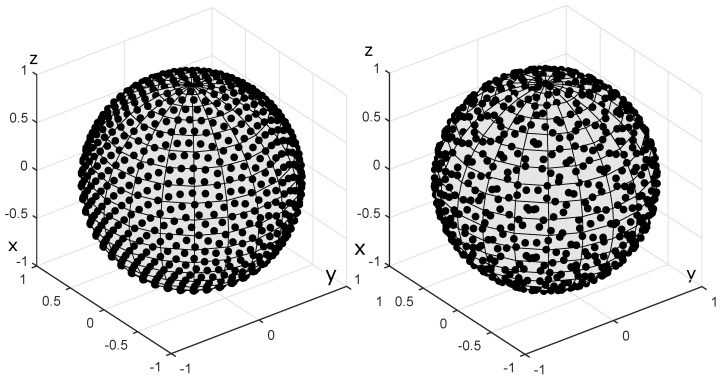
Sets of points on the unit sphere $S^{2}$. **Left**: Fibonacci lattice for N=1000. **Right**: Distribution based on the Sobol sequence for N=1000 with Skip = 0, Leap = 0.

## Data Availability

Not applicable.

## Appendix A. Implementation of the Equilibration Method

Note that the pair $X,\dot{X}$ uniquely describes the state of the system of charges. Let the vector of initial positions ${}^{0}X$ be random; assume that at t=0 the system is at rest: ${}^{0}\dot{X}=0$. Consider a generic time step $t_{n}\mapsto t_{n+1}$. The trial step is made by the explicit scheme:

(A1) $${}^{n+1}X^{\mathrm{trial}}={}^{n}X+\Delta t\cdot {}^{n}\dot{X},\;{}^{n+1}{\dot{X}}^{\mathrm{trial}}={}^{n}\dot{X}+\Delta t\cdot {}^{n}\ddot{X}.$$

Here, $\Delta t=t_{n+1}-t_{n}$. Using (19), we specify the expression for the current trial velocity:

(A2) $${}^{n+1}{\dot{X}}^{\mathrm{trial}}={}^{n}\dot{X}+\frac{\Delta t}{m}\left[{}^{n}F^{\tan\mathrm{gent}}-c\;{}^{n}\dot{X}\right].$$

The subsequent correction of the numerical solution is as follows. First, the trial position vectors at $t_{n+1}$ should be brought back to the unit sphere by the normalization:

(A3) $${}^{n+1}x_{i}={}^{n+1}x_{i}^{\mathrm{trial}}/∥{}^{n+1}x_{i}^{\mathrm{trial}}∥\;\mathrm{for}\;\mathrm{all}\;i=1,2,\ldots ,N.$$

Next, we discard the normal components of the velocities:

(A4) $${}^{n+1}{\dot{x}}_{i}=\left(1-{}^{n+1}x_{i}⊗{}^{n+1}x_{i}\right)\cdot {}^{n+1}{\dot{x}}_{i}^{\mathrm{trial}}\;\mathrm{for}\;\mathrm{all}\;i=1,2,\ldots ,N.$$

This completes the generic time step.

## Appendix B. Initial Approximation for the Uniform Distribution

The Fibonacci method allows creating the so-called Fibonacci point sets on the unit sphere $S^{2}$, also known as the Fibonacci lattice. The method consists of the following steps [48]. Let *N* be the required number of points on $S^{2}$, and let 0≤k≤N be a natural number. Then we set $\phi :=\pi (3-\sqrt{5})$; θ:=ϕ·k; $r:=\sqrt{\max(0,1-y^{2})}$, where y:=1−(k/(N−1))·2. The *x* and *z* coordinates of the Fibonacci points are computed as follows:

(A5) $$\left\{\begin{array}{c} x=r\cos(\theta );\\ z=r\sin(\theta ). \end{array}\right.$$

The second way of generating a set of points, close to uniform, is based on the pseudo-random Sobol sequence method. It utilizes a mapping of a uniform Sobol distribution from the unit square ${\left[0,1\right]}^{2}$ to the unit sphere $S^{2}$. This mapping is given by the following relations [49]:

(A6) $$\left\{\begin{array}{c} x=2\cos\left(2\pi \cdot x_{1}\right)\sqrt{x_{2}-x_{2}^{2}};\\ y=2\sin\left(2\pi \cdot x_{1}\right)\sqrt{x_{2}-x_{2}^{2}};\\ z=1-2x_{2}, \end{array}\right.$$

where $x_{1},x_{2}\in {\left[0,1\right]}^{2}$. Figure A1 shows the distributions obtained by the Fibonacci technique and the pseudo-random Sobol sequence for N=1000.
